# A Novel Technique for Validating Copy Number Variants and Its Application in Familial Exudative Vitreoretinopathy

**DOI:** 10.1155/humu/9556416

**Published:** 2026-09-12

**Authors:** Li-li Zhang, Zi-jia Zhao, Kai-xin Chen, Yu-qiao Ju, Feng-juan Gao, Yuan Zong, Qing Chang, Xin Huang, Ting Zhang, Ge-zhi Xu

**Affiliations:** ^1^ Eye Institute and Department of Ophthalmology, Eye and ENT Hospital, Fudan University, Shanghai, China, fudan.edu.cn; ^2^ NHC Key Laboratory of Myopia and Related Eye Diseases, Key Laboratory of Myopia and Related Eye Diseases, Chinese Academy of Medical Sciences, Shanghai, China, pumc.edu.cn; ^3^ Shanghai Key Laboratory of Visual Impairment and Restoration, Shanghai, China, fudan.edu.cn

**Keywords:** copy number variation, familial exudative vitreoretinopathy, next-generation sequence, quantitative PCR analysis, target enrichment

## Abstract

Inherited diseases have a strong genetic basis, with copy number variants (CNVs) playing a crucial role in their pathogenesis. However, the detection and validation of CNVs remain challenging. In this study, we introduce target enrichment polymerase chain reaction (tecPCR), a novel technique that integrates multiplex polymerase chain reaction (PCR) with next‐generation sequencing (NGS) for enhanced CNV validation, and applied it in familial exudative vitreoretinopathy (FEVR). Using NGS, 97 variants linked to FEVR were identified, including 91 SNVs and 6 CNVs. Of the CNVs, 66.67% (4/6) were validated both by quantitative PCR and tecPCR, yielding a positive predictive value of 100% (95% confidence intervals: 39.8%–100%) and negative predictive value of 100% (95% confidence intervals: 15.8%–100%) for tecPCR in this pilot validation. The validated FEVR genetic test showed a positivity rate of 53.07% (95/179). Moreover, the demographic and ocular features of the FEVR patients with CNVs were comparable to those with SNVs. This study broadens the mutation spectrum of FEVR and demonstrates that tecPCR provides exceptional specificity and sensitivity, making it a promising tool for genetic diagnostics.

## 1. Introduction

The widespread adoption of next‐generation sequencing (NGS), particularly exome sequencing, has identified numerous pathogenic variants, deepening our understanding of inherited diseases and uncovering their underlying mechanisms. NGS exhibits strong stability in detecting germline single‐nucleotide variants (SNVs), as validated by Sanger sequencing. However, accurately identifying germline copy number variants (CNVs) from hybridization‐captured NGS data remains challenging [1]. Numerous reviews highlight the low sensitivity, poor reproducibility, and risk of false positives in NGS‐screened CNVs, often necessitating additional gene dosage analyses.

Familial exudative vitreoretinopathy (FEVR) (OMIM#133780) is a rare, genetically heterogeneous disease affecting retinal angiogenesis, with a 0.11% incidence in newborns. Its retinal vascular anomalies cause incomplete or abnormal vascularization, leading to retinal folding, detachment, neovascularization, and potential blindness. More than 11 FEVR‐related genes have been identified, including *FZD4, NDP*, *LRP5, TSPAN12, ZNF408, KIF11, CTNNB1, JAG1*, and *ATOH7* [2]. However, most FEVR variants are classified as SNVs, with a notably low CNV detection rate [3]. The positive predictive value of NGS‐based CNV detection in FEVR varies significantly, from 10% to 80% for deletions and duplications [4]; however, the high throughput and resolution of NGS make it a promising tool for identifying novel disease‐related CNVs, particularly in cases without obvious pathogenic variants.

In this study, we designed a novel technique, target enrichment polymerase chain reaction (tecPCR) that combines multiplex polymerase chain reaction (PCR) with NGS, aimed at sensitively detecting copy number changes in multiple gene loci simultaneously in a single reaction. Moreover, we sought to use this approach to minimize nonspecific amplification, significantly enhancing CNV validation efficiency. Through our previously reported database of FEVR genetics and clinical phenotypes [5, 6], we compared the consistency of tecPCR with the widely accepted quantitative polymerase chain reaction (qPCR) method for CNV validation [7]. We also compared the clinical manifestations of participants with FEVR with SNVs and CNVs to enhance our understanding of phenotype‐genotype correlations, expand the variant spectrum of inherited diseases, and improve genetic diagnosis accuracy.

## 2. Materials and Methods

### 2.1. Participants

The study protocol was approved by the Ethical Oversight Committee of Fudan University Eye and ENT Hospital (Approval Number [2020] (2020119). All experiments were performed according to the tenets of the Declaration of Helsinki. Informed consent was obtained from all FEVR probands and family members.

Over 8 years (2017–2024), 179 FEVR probands and sporadic cases were diagnosed at Fudan University Eye and ENT Hospital, Shanghai, China, and underwent NGS for variant detection. Considering the limited number of identified individuals with CNVs FEVR probands and their family members with CNVs were assigned to the CNV group (*FZD4*: one proband and one family member; *TSPAN12*: one proband and one family member; *NDP*: two probands), whereas the probands with SNVs in the same genes as those in the CNV group were assigned to the SNV group (*FZD4*:37 probands; *TSPAN12*:29 probands; *NDP*: six probands).

### 2.2. Ocular Examinations

Standard ophthalmologic examinations were conducted for each family with FEVR and a sporadic case, including visual acuity tests, slit‐lamp biomicroscopy, intraocular pressure (IOP) measurement (noncontact tonometer), B‐ultrasound, fundus photography (Optos, Marlborough, Massachusetts), optical coherence tomography (OCT) (Heidelberg Engineering, Germany), optical coherence tomography angiography (OCTA) (Optovue Inc., Fremont, California; SVision Imaging, Ltd., China), and fundus fluorescein angiography (FFA) (Optos, Marlborough, Massachusetts). Axial length was measured using the IOL Master (Carl Zeiss Meditec, Inc., Jena, Germany). For statistical analysis, best corrected visual acuity (BCVA) was converted to LogMAR visual acuity (LogMAR = log [1/fractional visual acuity]). Counting finger vision was assigned LogMAR 2.0, hand motion vision LogMAR 2.3, light perception vision LogMAR 2.6, and no light perception vision LogMAR 3 [8]. When conducting an analysis of the ocular features, eyes with incomplete ocular data were excluded for further analysis.

#### 2.2.1. Sequencing and Primary Data Processing

NGS was conducted by Shanghai We‐Health Biomedical Technology Co., Ltd. Genomic DNA from peripheral leukocytes was subjected to exome capture using the XGen Exome Research Panel v1.0 (Integrated DNA Technologies, Inc., United States). Libraries were sequenced to generate 150 base pair paired‐end reads on an Illumina HiSeq4000 platform (Illumina, United States). We used fastp to generate quality control metrics of the raw FASTQ files (fastq.gz). Adapters were removed from the reads using cutadapt, and the first few bases of each read were trimmed using fastp. Unique molecular identifiers (UMIs) were extracted using Sentieon umi extract, followed by Sentieon umi consensus to generate consensus FASTQ files that collapse PCR duplicates and sequencing errors. The consensus reads were then aligned to the reference human genome (GRCh38) using Sentieon bwa mem. The resulting BAM files were sorted with Sentieon util sort. Note that no additional duplicate removal (e.g., Picard) was needed because the UMI‐based consensus step already mitigated duplicates.

#### 2.2.2. Variant Calling, Annotation, and Validation

Single‐nucleotide variants (SNVs) and small insertions/deletions (indels) were identified using Sentieon driver Haplotyper and GVCFtyper. Sample sex was inferred from genotype information derived from sex chromosome SNPs to facilitate sex‐specific copy number normalization. All potentially pathogenic SNVs and indels were validated by Sanger sequencing (PCR with ABI3730xl DNA Analyzer; Applied Biosystems, United States). The Ensembl Variant Effect Predictor16 (http://asia.ensembl.org/index.html), a functional annotation tool to predict the impact of genetic variants on gene/protein function by integrating multidatabase resources, was used to predict the variant pathogenicity with default settings and the GRCh38 reference genome. The National Centre for Biotechnology Information (https://www.ncbi.nlm.nih.gov/), 1000 Genomes Database (http://www.1000genomes.org/), gnomAD (https://gnomad.broadinstitute.org/), and Exome Sequencing Project (https://evs.gs.washington.edu/EVS/) were searched to determine if each variant had been previously reported.

#### 2.2.3. Genome‐Wide CNV Analysis

Genome‐wide copy number and loss‐of‐heterozygosity (LOH) analysis was performed using CNVkit. The robustness of this analysis is underpinned by our custom sequencing panel design, which, in addition to the gene‐specific probes, includes a comprehensive set of baits spaced at approximately 0.3‐Mb intervals across the entire genome. This design enables accurate inference of copy number alterations by comparing the sequencing depth in target regions against this genome‐wide background. The resulting copy number and LOH profiles were visualized and interpreted using the Nexus Copy Number software (BioDiscovery, United States). Regions with normalized ratios ≥ 1.2 and ≤ 0.8 were classified as duplications and deletions, respectively. The average sequencing depth of target regions was required to be ≥ 1000x. For tecPCR normalization, reference regions were selected from stable‐copy‐number loci within the panel and excluded regions with repetitive sequences, high coverage variability, abnormal GC content, or potential genomic instability. The pathogenicity of identified CNVs was interpreted according to the joint consensus recommendations of the American College of Medical Genetics and Genomics and the Clinical Genome Resource. This evidence‐based framework assigns a classification of pathogenic, likely pathogenic, uncertain significance, likely benign, or benign by evaluating multiple lines of evidence, including the CNV′s genomic content, overlap with dosage‐sensitive genes, population frequency, and available inheritance data. For assessing the population rarity of the target CNV, we queried the Database of Genomic Variants (DGV) (accessed on 1 December 1, 2025) Gold Standard dataset with the GRCh38/hg38 reference genome assembly.

### 2.3. Verification of CNVs

qPCR and tecPCR techniques were performed on suspected CNVs from NGS data and healthy controls.

#### 2.3.1. qPCR

Primers for qPCR validation were designed based on CNV fragment length and location, verified using Primer3Plus (http://www.primer3plus.com/) and in silico PCR (http://genome.ucsc.edu/cgi-bin/hgPcr). For each patient, amplicons within the CNVs, control amplicons upstream or downstream of the CNVs, and control amplicons on autosomes and the X chromosome were designed for multiple rounds, and the primers for the final successful experiments are listed in Tables S1, S2, S3, S4, S5, S6. After amplification, relative DNA quantification was analyzed using the 2^−△△Ct^ method, with GAPDH used as the normalization control.

#### 2.3.2. tecPCR

**Figure 1:**
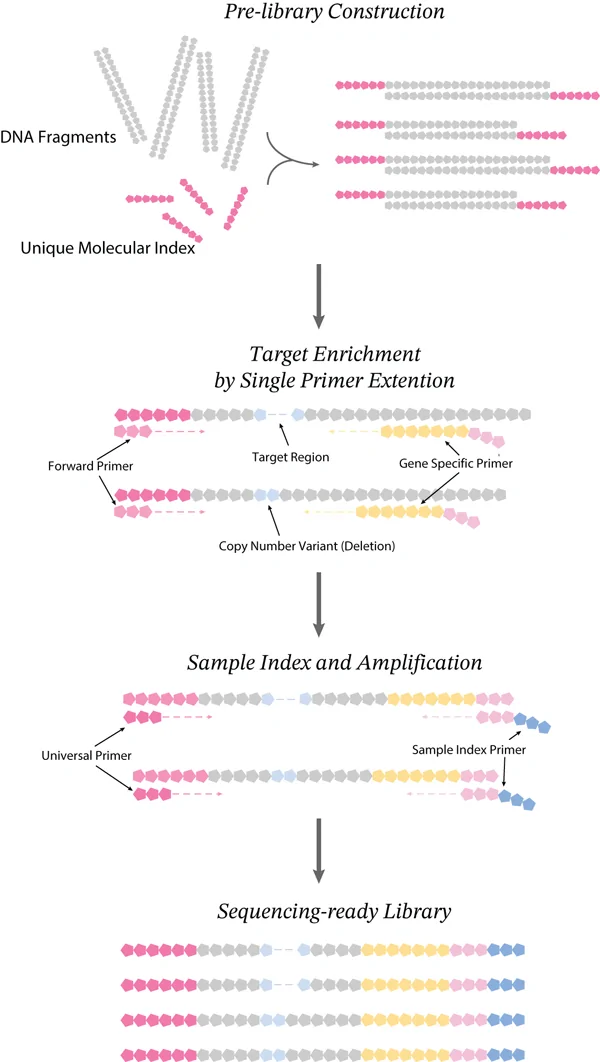
Technological roadmap of tecPCR for CNV validationGenomic DNA is first fragmented and ligated with unique molecular identifiers (UMIs) to generate a prelibrary. Target enrichment is subsequently performed using gene‐specific PCR primers. Following sample indexing and amplification, the libraries undergo quality control and are prepared for next‐generation sequencing (NGS).

After genomic DNA extraction and fragmentation, a short unique nucleotide sequence, UMI, was ligated to the ends of the DNA fragments to prepare a prelibrary. Target enrichment was then performed using gene‐specific primers (Table S7). After sample indexing and amplification, labeled libraries were quantified, quality‐checked, and subjected to high‐throughput sequencing. The tecPCR technology roadmap is shown in Figure 1. Using bioinformatics software, sequencing data were analyzed as follows:

CNVtarget=dpST/dpSRdpCT/dpCR.

where dpST is the depth of the target region of the sample to be tested, dpSR is the depth of the reference gene region of the sample to be tested, dpCT is the depth of the target region in the control sample, and dpCR is the depth of the reference gene region in the control sample.

### 2.4. Statistics

Statistical analyses were conducted using SPSS Version 23.0 (IBM Corp., Armonk, New York, United States), with *p* < 0.05 considered statistically significant. Generalized estimating equation (GEE) models were performed as sensitivity analyses. Bar graphs represent the mean ± standard deviation (SD). Demographic and ocular features were compared between the CNV and SNV groups. Data normality was assessed using the Shapiro–Wilk test. Student′s *t* *-*test, Mann–Whitney *U*, and chi‐square tests were used based on the data type.

## 3. Results

### 3.1. Genetic Analysis

**Figure 2:**
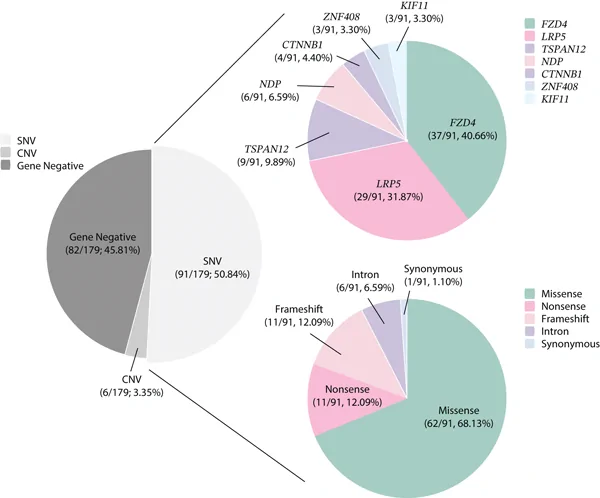
NGS results of copy number variation of FEVR and subgroup analysis The left pie chart shows the proportions of SNVs and CNVs identified by next‐generation sequencing. The left pie chart shows the proportions of SNVs and CNVs among all genetically diagnosed families. The upper right pie chart illustrates the distribution of disease‐causing genes among SNV‐positive families, including FZD4, LRP5, TSPAN12, NDP, CTNNB1, ZNF408, and KIF11. The lower right pie chart summarizes the molecular diagnosis of SNVs, including missense, nonsense, frameshift, intronic, and synonymous variants.

Among the 179 unrelated families, NGS detected pathogenic variants in 97 families, comprising 91 SNVs and six CNVs (Figure 2). All SNVs were validated by Sanger sequencing. The SNVs included 37 (40.66%) in *FZD4*, 29 (31.87%) in *LRP5*, nine (9.89%) in *TSPAN12*, six (6.59%) in *NDP*, four (4.40%) in *CTNNB1,* three (3.30%) in *ZNF408,* and three (3.30%) in *KIF11* (Figure 2). Among these, 62 (68.13%) were missense variants, 11 (12.09%) were nonsense variants, 11 (12.09%) were frameshift variants, six (6.59%) were intron variants, and one (1.10%) was a synonymous variant (Figure 2).

**Figure 3:**
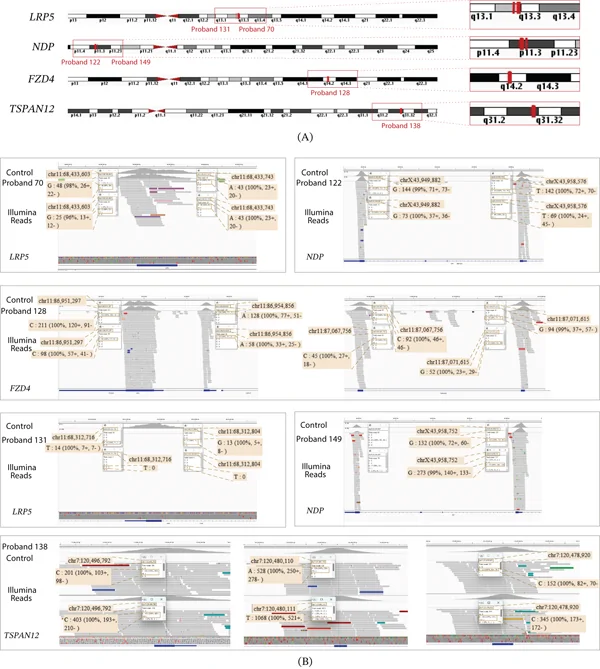
The localization of different CNV mutations in visualization analysis tools. Integrative genomics viewer shows four CNVs detected in FEVR families. Panel (A) indicates the chromosomal positions of the CNVs. Panel (B) illustrates NGS read counts across the affected regions in the probands and controls. The label includes CNV positions, nucleotide type, and read counts. CNV analysis is based on comparisons of read depth between the control and proband.

The six NGS‐detected CNVs in the six FEVR families were considered pathogenic, as no other pathogenic SNVs or polymorphic variants associated with FEVR causative genes were found. The integrative genomics viewer and details of these CNVs are shown in Figure 3, Figure S1, and Table 1, respectively. All the CNVs are novel, with two located in *LRP5*, two in *NDP*, one in *FZD4,* and one in *TSPAN12* (Table 1). Regarding the variant type, four were heterozygous deletions, one was a homozygous deletion, and one was a heterozygous duplication (Table 1).

**Table 1:** Copy number variants screened by next‐generation sequencing.

Patient No.	Sex/onset age	Fragment length	Genomic location (hg38)	Chromosome region	Variant type	OMIM gene	Effect	Heredity	ACMG	Reference
70	F/7	238 bp	chr11:68433601‐68433838	11q13.2q13.2	Heterozygous	*LRP5*	Exon 18 deletion	De novo	LP	Novel
122	F/3	9.12 Kbp	chrX:43949798‐43958913	Xp11.3p11.3	Heterozygous	*NDP*	Exons 2–3 deletion	De novo	LP	Novel
128	F/8	120.47 Kbp	chr11:86951141‐87071615	11q14.2q14.2	Heterozygous	*FZD4*	Exons 1–2 deletion	Mother	P	Novel
131	M/4	92 bp	chr11:68312714‐68312805	11q13.2q13.2	Homozygous	*LRP5*	Exon 1 deletion	De novo	P	Novel
138	F/3	3.61Kbp	chr7:120478884‐120840030	7q31.3q31.3	Heterozygous	*TSPAN12*	Exons 2–4 deletion	Mother	P	Novel
149	M/2	0.44Kbp	chrX:43958471‐43958913	Xp11.3p11.3	Heterozygous	*NDP*	Exon 2 duplication	De novo	VUS	Novel

Abbreviations: ACMG, American College of Medical Genetics and Genomics; OMIM, Online Mendelian Inheritance in Man.

### 3.2. CNV Verification

**Figure 4:**
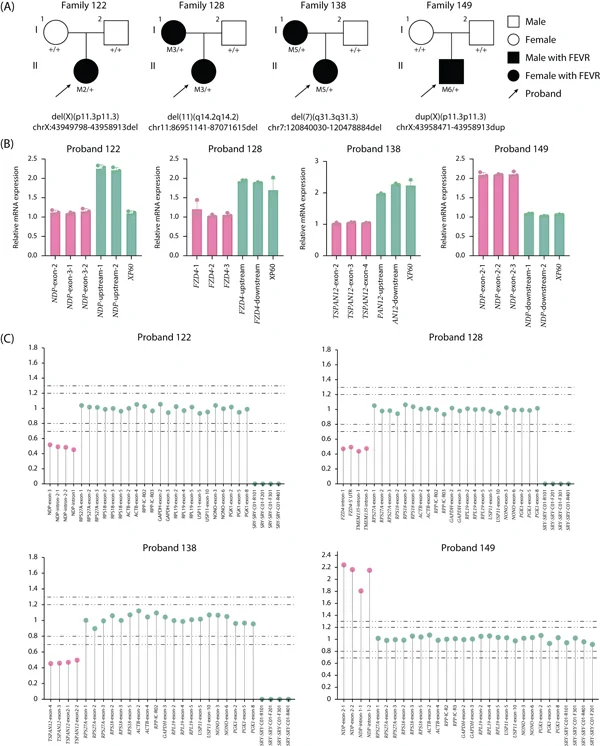
Pedigree cosegregation and CNVs verification by qPCR and tecPCR. (A) Pedigree cosegregation analysis of the four validated CNVs identified in Families 122, 128, 138, and 149. (B) qPCR confirms four WES‐based CNVs are authentic. Four CNVs include three deletions (Families 122, 128, and 138) and one duplication (Family 149), whereas the exon 18 deletion in LRP5 (Family 70) and the exon 1 deletion in LRP5 (Family 131) were not confirmed and were considered false‐positive CNVs. (C) tecPCR confirms four WES‐based CNVs are authentic. Validation of the same CNVs using tecPCR. The horizontal axis indicates the genomic positions of the target amplicons, and the vertical axis represents the copy number ratio between the patient and control samples. tecPCR results correspond with qPCR and confirm the three deletions and one duplication identified by NGS. CNV, copy number variant; qPCR, quantitative polymerase chain reaction; tecPCR, target enrichment polymerase chain reaction.

Four CNVs detected by NGS (Families 122, 128, 138, and 149) were validated by qPCR (Figure 4A,B). The other two were false positives (Family 70: exon 18 deletion in *LRP5* and Family 131: exon 1 deletion in *LRP5)* (Figure 4A,B). Segregation analysis indicated that the exons 1–2 deletion in *FZD4* (Family 128) and exons 2–4 in *TSPAN12* (Family 138) were maternally inherited, whereas the exons 2–3 deletion in *NDP* (Family 122) and the exon 2 duplication in *NDP* (Family 149) were de novo variants (Figure 4A).

tecPCR results strongly aligned with qPCR, which successfully detected three deletions (Families 122, 128, and 138) and one duplication (Family 149) (Figure 4C). tecPCR results strongly aligned with qPCR, which successfully detected three deletions (Families 122, 128, and 138) and one duplication (Family 149) (Figure 4C). tecPCR also validated that the two CNVs found in Families 70 and 131 were false positives. This yielded a PPV of 100% (95% CI: 39.8%–100%) and a NPV of 100% (95% CI: 15.8%–100%). Although both qPCR and tecPCR successfully verified CNVs, qPCR required multiple rounds of primer optimization and design, whereas tecPCR only required one experiment. In addition, the GC content of qPCR primers used for breakpoint detection was significantly higher than that of tecPCR primers (Figure S2). For some qPCR primers, the GC content even exceeded 60%.

None of the deletions or duplications detected in this study were found in healthy controls by either qPCR or tecPCR. In addition to its absence in our internal control cohort, these CNVs showed no overlap with benign variants in the DGV Gold Standard dataset. Among 179 FEVR probands and sporadic cases, 95 carried pathogenic or likely pathogenic variants in known FEVR‐associated genes, yielding an overall molecular diagnostic rate of 53.07%.

### 3.3. Clinical Manifestation of Participants With FEVR Carrying CNVs

**Figure 5:**
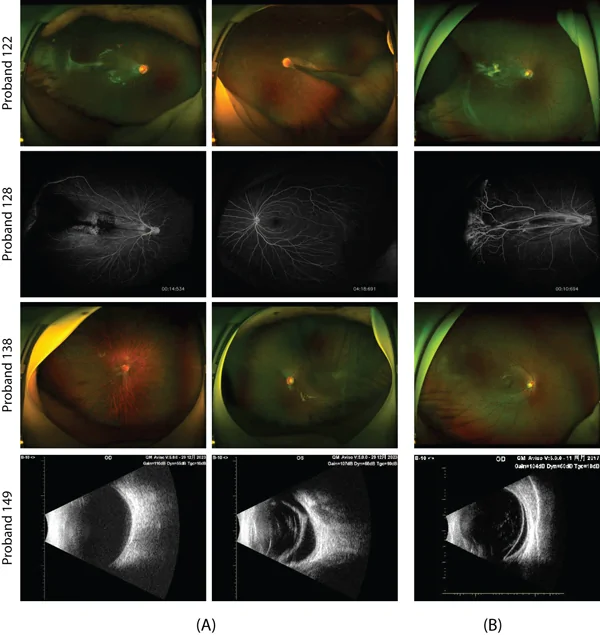
Ocular phenotypes of participants with FEVR carrying CNVs and SNVs. (A) Ocular phenotypes of FEVR probands with confirmed CNVs. Typical FEVR manifestations include peripheral retinal avascularity, retinal folds, retinal detachment, vitreoretinal traction, and retinal neovascularization. (B) Ocular phenotypes of FEVR probands with confirmed SNVs. CNV, copy number variants; SNV, single‐nucleotide variant.

The ocular manifestations of the four probands with CNVs are shown in Figure 5A. Proband 122 (*NDP* exons 2–3 heterozygous deletion) sought treatment for leukocoria in the left eye. Given the peripheral nonperfusion area, reduced retinal arteriolar angle, and temporal vitreoretinal traction in her right eye, the patient was highly suspected of having FEVR. She underwent cataract surgery and genetic testing. After the cataract surgery, a retinal fold was observed in the left eye. The fundus of proband 128 (*FZD4*, exons 1–2 heterozygous deletion) was highly asymmetric. A peripheral nonperfusion zone, retinal fold, straightened vessel branching, retinal neovascularization, and vascular leakage were observed in the fundus of the right eye. In contrast, only a peripheral nonperfusion zone was observed in the left eye. Fundus photography of proband 138 (*TSPAN12* exons 2–4 heterozygous deletion) is shown in Figure 5A. Proband 149 (*NDP* exon 2 heterozygous duplication) presented with unilateral retinal detachment.

We compared the demographic and ocular features between the CNV and SNV groups with variants in *FZD4, TSPAN12,* and *NDP* (Tables 2 and 3), analyzing the eyes of the FEVR probands carrying SNVs or CNVs, and family members with CNVs. The mean age at onset, proportion of female participants, and genotype distribution were similar between the CNV and SNV groups (all *p* > 0.05, Table 2). BCVA, axial length, IOP, retinal fold occurrence, and FEVR stage were similar between the two groups (all *p* > 0.05, Table 3). Sensitivity analyses using GEE models yielded results consistent with the original analyses. The ocular features in the CNV group were not specific, with typical FEVR manifestations, including retinal fold, vitreoretinal traction, peripheral avascular area, retinal neovascularization, and retinal detachment, also observed in the SNV group (Figure 5B).

**Table 2:** Demographic information of FEVR eyes across different groups.

	SNV (*n* = 52)	CNV (*n* = 6)	*p* value
Age	12.95 ± 10.85	12.50 ± 13.95	0.93
Sex (male/female)	28/24	1/5	0.19
Genotype			
*FZD4*	37	2	0.16
*TSPAN12*	9	2	
*NDP*	6	2	

Abbreviations: CNV, copy number variant; SNV, single‐nucleotide variant.

**Table 3:** Ocular features of FEVR eyes across different groups.

	SNV (*n* = 91)	CNV (*n* = 12)	*p* value
BCVA (LogMAR), middle, range	0.52, 0–3	0.85, 0–2.3	0.61
Retinal detachment	36/91	1/12	0.052
Optic drag	29/91	5/12	0.53
Retinal fold	17/91	3/12	0.70
Axial length	23.73 ± 1.87	24.22 ± 0.62	0.72
IOP	13.79 ± 3.63	12.70 ± 1.70	0.68
FEVR stage			
Stage 1	54	10	0.52
Stage 2	11	1	
Stage 3	8	0	
Stage 4	11	1	
Stage 5	7	0	

Abbreviations: BCVA, best corrected visual acuity; CNV, copy number variant; IOP, intraocular pressure; SNV, single‐nucleotide variant.

## 4. Discussion

This study introduces tecPCR, a novel CNV validation technique consistent with the established qPCR method. NGS identified pathogenic variants in 97 of the 179 FEVR families, including six with potential novel CNVs. Four of the six CNVs were validated, expanding the FEVR variant spectrum and emphasizing the need for subsequent CNV validations following NGS identification. Genotype–phenotype analysis revealed no statistically significant differences in demographics. No statistically significant differences in ocular characteristics were observed between the CNV and SNV groups in this limited cohort. NGS is a powerful tool for diagnosing inherited diseases, primarily for detecting SNVs. Although not typically recommended for CNV detection, it can simultaneously identify SNVs and CNVs [9]. When screening for CNVs with NGS, complementary methods such as qPCR are essential. This is due to uneven exon capture and fluctuating sequencing depth, which can lead to false positives, particularly for short CNVs of a few hundred or tens of base pairs confined to the same exon. This aligns with our findings, where NGS detected a heterozygous 238‐bp deletion in exon 18 of *LRP5* (Family 70) and a homozygous 92‐bp deletion in exon 1 of *LRP5* (Family 131), later confirmed as false positives. It is worth noting that in proband 131, the GC content in the CNV region was high and the read count was 0. Even in normal controls, the read count in this region is very low. Therefore, in this case, there is a high likelihood of false positives. Despite these limitations, several studies have successfully used NGS to detect CNVs [1, 10–12]. Luo et al. screened eight CNVs using NGS in 406 FEVR probands without definite pathogenic variants, confirming five of them [13]. Huang et al. detected four CNVs in *FZD4* and three CNVs in *NDP* based on NGS data, all confirmed by qPCR [14, 15]. NGS has also been used to detect CNVs in other inherited retinal diseases and is not limited to FEVR. Hiraoka et al. confirmed eight CNVs found in participants with retinitis pigmentosa using NGS [12]. In our study, NGS detected six CNVs in unrelated families, with 66.7% (4/6) confirmed. These findings highlight NGS′s potential for CNV detection and the importance of further validation to avoid false positives.

tecPCR, a technique that combines multiplex PCR with NGS, can sensitively detect copy number changes at multiple gene loci in a single reaction, improving detection efficiency. The “target” refers to the DNA segment of interest. The “enrichment” occurs as PCR amplifies the target. Specific primers replicate the target sequence multiple times, increasing its proportion in the sample and distinguishing it from the rest of the DNA.

Compared with traditional qPCR, tecPCR could reduce nonspecific amplification typically seen in qPCR. The unidirectional primers utilized in tecPCR have longer sequences than the bidirectional primers employed in qPCR, which endow tecPCR with enhanced specificity. The addition of UMIs [16] and single‐end specific primer‐mediated amplification in tecPCR distinguish false positives from genuine variants. In our results, NGS of tecPCR products effectively identified CNVs, and no tecPCR experiments showed nonspecific amplification. In addition, the gene‐specific primers designed for specific exons/introns in tecPCR can be used repeatedly in the detection of different CNVs, avoiding the need to design specific primers for the breakpoints, upstream and downstream of different nucleotide fragments, which are essential processes in traditional qPCR. Therefore, tecPCR represents a promising CNV validation approach that may facilitate targeted copy‐number analysis in research and diagnostic settings, effectively eliminating the necessity of repeated primer design steps. In the present study, although we achieved success in all qPCR experiments for CNVs, specific primer designs were used in multiple rounds for all different CNVs, whereas tecPCR experiments were successful on the first try, which made qPCR less efficient than tecPCR. Besides, when the GC content of a CNV is high, tecPCR, not restricted by specific CNV regions, can avoid the high‐GC sequence when designing primers. However, it may be difficult for qPCR to avoid these high‐GC regions. In this study, for breakpoints, the GC content of qPCR primers was higher than that of tecPCR primers, and for some qPCR primers, it even exceeded 60%. An excessively high‐GC content may prevent primers from effectively binding to the template, resulting in failed amplification. Additionally, primers with high‐GC content may increase nonspecific binding, leading to nonspecific amplification. In this regard, the primer design of tecPCR is more convenient. Moreover, the consistency between qPCR and tecPCR results validated the reliability of tecPCR, suggesting that tecPCR is a reliable technique for CNV validation, and tecPCR kits tailored for target genes could enhance CNV identification and expand the range of relevant diseases.

Therefore, tecPCR method joins a growing family of CNV validation approaches, such as quantitative real‐time PCR (qPCR), droplet digital polymerase chain reaction (ddPCR), digital Multiplex Ligation‐dependent Probe Amplification (dMLPA). qPCR and ddPCR remain widely used and highly reliable methods for targeted CNV confirmation, with ddPCR providing particularly accurate molecular quantification. Similarly, dMLPA offers robust multiplex CNV assessment and has been extensively validated in clinical diagnostics. Although current technology typically relies on predefined, commercial probe sets, which can limit its flexibility for interrogating custom genomic regions. In contrast, tecPCR leverages standard PCR primer design principles, offering greater adaptability for individual laboratories to rapidly design and validate CNVs in specific candidate genes. However, it is important to acknowledge that for large‐scale, standardized screening of a fixed gene set, dMLPA remains a highly efficient alternative.

Previous studies demonstrated that CNVs in *NDP* are associated with more severe ocular phenotypes [15]. Li et al. reported three patients with *NDP* CNVs (two were exon 2 deletions and one whole‐gene deletion), all developing total retinal detachment at birth or shortly after [15]. Arai et al. described a family with an exon 2 deletion in the *NDP* gene, presenting with shallow anterior chambers, corneal opacification, cataracts, and retinal detachment [17]. Consistent with these findings, proband 149, with an *NDP* exon 2 duplication identified in this study, presented with unilateral total retinal detachment. *NDP*‐related retinopathy is generally an X‐linked recessive disorder. However, most female carriers of *NDP* variants also exhibited mild clinical features of FEVR [5, 18]. Proband 122, a female CNV carrier of *NDP* (exons 2–3 deletion), showed congenital cataracts, vitreoretinal traction, and retinal folds. These findings suggest that *NDP* is a dosage‐sensitive gene, with both overexpression and underexpression affecting retinal development.

CNVs in *TSPAN12* and *FZD4* are linked to mild phenotypes and asymmetry [14, 19], respectively. Zhang et al. identified a heterogeneous *TSPAN12* deletion in one family with mild phenotypes, including straightened blood vessels, a falciform retinal fold, and avascular zones [19]. Consistent with their findings, proband 138 (*TSPAN12* exons 2–4 deletion) exhibited a mild phenotype, with a reduced retinal arterial angle in the right eye. Li et al. identified four heterozygous whole‐gene deletions of *FZD4* across four families [14], all presenting with unilateral retinal folds. Similarly, proband 128 (*FZD4* exon 1–2 deletion) exhibited a unilateral retinal fold. Current studies on genotype‐phenotype correlations in FEVR are limited, and our findings contribute to understanding clinical characteristics associated with CNV‐related FEVR.

Although affecting a larger portion of the genome, the CNVs in this study did not result in more severe FEVR phenotypes. Ocular features (BCVA, axial length, IOP, retinal fold, and FEVR stage) in participants with CNV were similar to those of patients with SNVs. A previous study found that FEVR severity in patients with CNVs is no greater than that in patients with SNVs [20]. Not limited to FEVR, there is currently no consensus on whether CNVs cause more severe phenotypes than SNVs. Although types differ in pathogenic mechanisms, SNVs can lead to amino acid sequence changes from nonsynonymous variants, protein truncation, or altered growth from splice variants [21]. For CNVs, gene dosage imbalances due to integer‐fold changes can occur. Protein truncation or growth from deletions or duplications in CNVs may resemble the effects of SNVs caused by variants affecting mRNA splicing. Advances in NGS and bioinformatics tools have enhanced our ability to assess SNV pathogenicity. However, accurate CNV identification remains challenging, hindering our understanding and assessment of their pathogenicity and phenotype‐genotype correlation. Therefore, new methods for CNV validation are urgently needed. tecPCR, reported in this study, is a promising candidate for CNV identification.

Our study has some limitations. The small number of CNV carriers in our study limits our understanding of the phenotypic effects of CNVs. Although no statistically significant differences in disease severity were observed between CNV and SNV carriers, larger studies are required to more robustly evaluate potential genotype–phenotype differences. Our preliminary assessment of tecPCR′s accuracy is limited by small cohort size and lack of an independent gold standard for definitive case‐control classification; a future prospective multicenter study with statistically powered sample size is thus required to robustly validate tecPCR performance.

## 5. Conclusions

In summary, we introduced tecPCR, a novel technique for amplifying target genes and validating CNVs with enhanced sensitivity. Compared with traditional methods, tecPCR offers improved detection efficiency and avoids nonspecific amplification. Developing tecPCR kits for specific genetic diseases or target genes could streamline CNV detection and eliminate the need to design specific primers, as traditional PCR requires.

## Author Contributions

Conceptualization and project administration: X.H. and T.Z.; funding acquisition: Y.Z., Q.C., G.X., and T.Z.; investigation, resources, and validation: L.Z. and Y.J.; writing—original draft: L.Z., K.C., and Y.J.; writing—review and editing: F.G. and Y.Z. All authors have read and agreed to the published version of the manuscript. L.Z., Z.Z., and K.C. contributed equally to this work.

## Funding

This study was supported by the National Natural Science Foundation of China (NSFC 82201204, 82171078), Shanghai Hospital Development Center Foundation (SHDC12023116), and the 2024 Clinical Scientists Programme of Shanghai Medical School.

## Ethics Statement

The study protocol was approved by the Ethical Oversight Committee of Fudan University Eye and ENT Hospital (Approval Number [2020] (2020119). All experiments were performed according to the tenets of the Declaration of Helsinki. Informed consent was obtained from all FEVR probands and family members.

## Conflicts of Interest

The authors declare no conflicts of interest.

## Supporting information


**Supporting Information** Additional supporting information can be found online in the Supporting Information section. The Supporting Information material includes two Supporting Information figures and seven Supporting Information tables that provide additional results and supporting information for the main analyses.

## Data Availability

The sequencing data supporting the findings of this study have been deposited in Mendeley Data under reserved doi:10.17632/ypndp3xyw2.1 (https://data.mendeley.com/).
