# Supplementary material for: A Novel Technique for Validating Copy Number Variants and Its Application in Familial Exudative Vitreoretinopathy
Source: Hum Mutat. 2026 Sep 12;2026:9556416. doi: 10.1155/humu/9556416 (PMC13570494; doi:10.1155/humu/9556416)
Supplement: Supplementary file 1 [file HUMU-2026-9556416-s001.docx]

**Supplementary material**

**Supplementary Figure 1 Integrative genomics viewer**

Integrative genomics viewer of the upstream and/or downstream of the copy number variants.

**Supplementary Figure 2 A comparative analysis of the GC content of primers employed in qPCR and tecPCR**

QPCR, quantitative polymerase chain reaction; tecPCR, target-enrichment polymerase chain reaction.

| **Table. S1. primer information designed for qPCR for family 70** | | | |
| --- | --- | --- | --- |
| **Amplicon** | **Sequence** | **Genomic region (hg38)** | **Remark** |
| *LRP5-*exon-18-1 | 5’- TGCCACAGGGGAGATCGAC -3’ 5’- CTCCTCGTCGCTCTGGTCA -3’ | chr11:68433636-68433717 | Exon18 (deletion region) |
| *LRP5-*exon-18-2 | 5’- ATGACCAGAGCGACGAGGAG -3’ 5’- GTCCACACACTGACCCCG -3’ | chr11:68433698-68433774 | Exon18 (deletion region) |
| *LRP5-*exon-18-3 | 5’- GTCAGTGTGTGGACCTGCG -3’ 5’- ACAGTCCGCCTCGTCTGA -3’ | chr11:68433761-68433834 | Exon18 (deletion region) |
| *LRP5-*upstream | 5’- GTATGCACAACAACGGGCAG -3’ 5’- GTCCAGGGTGTAGTGTGAGG -3’ | chr11:68413902-68413990 | Upstream of deletion region |
| *LRP5-*downstream | 5’- ACGTGTGTGACAGCGACTAC -3’ 5’- AGGGGTCTGAGTCCGAGTTC -3’ | chr11:68448859-68448939 | Downstream of deletion region |

| **Table. S2. primer information designed for qPCR for family 122** | | | |
| --- | --- | --- | --- |
| **Amplicon** | **Sequence** | **Genomic region (hg38)** | **Remark** |
| *NDP-*exon-2 | 5’- CAGACAGTAAAACGGACAGCTC -3’  5’- AAATGCTCTCCTCACAGAGACC -3’ | chrX:43958440-43958581 | Exon2 (deletion region) |
| *NDP-*exon-3-1 | 5’- TGTCGTTCAGCACTGTCCTC -3’  5’- CAGTGCCTTCAGCTTGGAAG -3’ | chrX:43949883-43949965 | Exon3 (deletion region) |
| *NDP-*exon-3-2 | 5’- CCTTTTCTTTCCCCAGATGGTG -3’  5’- AGTGCTGAACGACACCAAAG -3’ | chrX:43949952-43950042 | Exon3 (deletion region) |
| *NDP-*upstream-1 | 5’- GGAAGATGAACAGTAAGTCTGCTTACC -3’  5’- GTGTTTTAGTCATATAAGGCCATGGCT -3’ | chrX:44084366-44084612 | Upstream of deletion region |
| *NDP-*upstream-2 | 5’- TCTGACTCTGACAGAGACCCTCA -3’  5’- ATGCCAACACCGAGCCAATTAAG -3’ | chrX:44095590-44095696 | Upstream of deletion region |

| **Table. S3. primer information designed for qPCR for family 128** | | | |
| --- | --- | --- | --- |
| **Amplicon** | **Sequence** | **Genomic region (hg38)** | **Remark** |
| *FZD4*-1 | 5’- TTTCCCCTCCATTCCGCAC -3’ 5’- CCAAGTGTGGCCGATCTCAT -3’ | chr11:87037924-87038102 | Whole gene (deletion region) |
| *FZD4*-2 | 5’- AAGTGGGAAATGTTGGTTGGAT -3’ 5’- TGAAGCGGATTGTAGGATCTCAG -3’ | chr11:87067645-87067771 | Whole gene (deletion region) |
| *FZD4*-2 | 5’- TCATGGACTCCTGGCTTTGG -3’ 5’- TGGGGTAGGGGACAGGTAAT -3’ | chr11:87071545-87071667 | Whole gene (deletion region) |
| *FZD4-*upstream | 5’- CACTGGCAAAAATCCCTCGG -3’ 5’-GCGTTTGGGAGAGGATGAAGA -3’ | chr11:86446466-86446598 | Upstream of deletion region |
| *FZD4-*downstream | 5’- ACCGGTTTGCCATTAGGGAG -3’ 5’- CCGAGAAGCTATGGGTGCAT -3’ | chr11:88149799-88149918 | Downstream of deletion region |

| **Table. S4. primer information designed for qPCR for family 131** | | | |
| --- | --- | --- | --- |
| **Amplicon** | **Sequence** | **Genomic region (hg38)** | **Remark** |
| *LRP5-*exon-1 | 5’- AGCCTAGAGGCACTTTTAGAGG  AGTGTGTCTGTTT -3’ 5’- AGAACATGTGTAGCGTTTCAC  GACTGCTTAAGTTT -3’ | chr11:68311714-68313805 | Exon1 (deletion region) |
| *LRP5-upstream* | 5’- TGGTGGGTTGTATTGCCGAA -3’ 5’- CAGCCAGAGTTGAGCACAGT -3’ | chr11:68171568-68171683 | Upstream of deletion region |
| *LRP5-*downstream | 5’- ACGTGTGTGACAGCGACTAC -3’ 5’- AGGGGTCTGAGTCCGAGTTC -3’ | chr11:68448859-68448939 | Downstream of deletion region |

| **Table. S5. primer information designed for qPCR for family 138** | | | |
| --- | --- | --- | --- |
| **Amplicon** | **Sequence** | **Genomic region (hg38)** | **Remark** |
| *TSPAN12-*exon-2 | 5’- CCCATGCAAGAAGCAACAGATT -3’ 5’- TCATCCGGTCATGATTGCTGT -3’ | chr7:120838776-120838873 | Exon2-4 (deletion region) |
| *TSPAN12-*exon-3 | 5’- AGCGTAAAGTTGGGGACACC -3’ 5’- TCTCTGGCCATCCGTAGTGA -3’ | chr7:120851102-120851254 | Exon2-4 (deletion region) |
| *TSPAN12-*exon-4 | 5’- CATGCCCTTTGGCGCATTAT -3’ 5’- TGCTCTACGCCCTCAATCTG -3’ | chr7:120856634-120856707 | Exon2-4 (deletion region) |
| *TSPAN12-*upstream | 5’- AGCACACCTTATCGTCTCAAGTCA -3’ 5’- TGTGGGATCTCAAAAGCTTGC -3’ | chr7:121371287-121371380 | Upstream of deletion region |
| *TSPAN12-*downstream | 5’- TGCCAAAAACCATAGCAGGGA -3’ 5’- ATCACCGGAACAGGTAGAGC -3’ | chr7:120732937-120732982 | Downstream of deletion region |

| **Table. S6. primer information designed for qPCR for family 149** | | | |
| --- | --- | --- | --- |
| **Amplicon** | **Sequence** | **Genomic region (hg38)** | **Remark** |
| *NDP-*exon-2-1 | 5’- TCGAGGGTCCGAGTCCATTA -3’ 5’- TCTATGCTCTCCCTGCTGGT -3’ | chrX:43958535-43958615 | Exon2 (duplication region) |
| *NDP-*exon-2-2 | 5’- AGGATCCAGTCCCGTTCAAG -3’ 5’- TCCTGGGTTCCATTAGTGGTT -3’ | chrX:43958723-43958918 | Exon2 (duplication region) |
| *NDP-*exon-2-3 | 5’- ACCAGCAGGGAGAGCATAGA -3’ 5’- GAAAGCTTCAGCCCGATCCT -3’ | chrX:43958596-43958770 | Exon2 (duplication region) |
| *NDP-*downstream-1 | 5’- GCTTACCTTAGGGGAACGCA -3’ 5’- AGCATTTGGAAGTAACAGGACC -3’ | chrX:43973298-43973379 | Upstream of duplication region |
| *NDP-*downstream-2 | 5’- TCTCGATCTTCATACACACAGGA -3’ 5’- ACTGGCCCACGAAAAGTTCA -3’ | chrX:44178370-44178450 | Upstream of duplication region |

| **Table. S7. primer information designed for tecPCR** | | | | |
| --- | --- | --- | --- | --- |
| **Sample** | **Amplicon** | **Sequence** | **Primer direction** | **Genomic region (h38)** |
| Family 70 | *LRP5*-exon-18-1 | ACACGACGCTCTTCCGATCTATACAGTCGATCTCCCCTGTGGCACAT | Reversed | chr11:68433633-68433659 |
|  | *LRP5*-exon-18-2 | ACACGACGCTCTTCCGATCTAGACTGTCAGGACCGCTCAGACGAG | Forward | chr11:68433801-68433825 |
|  | *LRP5*-intron-18 | ACACGACGCTCTTCCGATCTAAACTCCTGACCTAATGCAGGAAGGTGAGATAGAC | Reversed | chr11:68434054-68434088 |
| Family 122 | *NDP-*exon-3 | ACACGACGCTCTTCCGATCTATGAACCAAAAGAAATTGTACCAGAGAAAGGAGATG | Forward | chrX:43949569-43949604 |
|  | *NDP*-intron-2-1 | ACACGACGCTCTTCCGATCTTTACTCTCAATACTTCACATTTTAGCTTCTGCCATG | Forward | chrX:43953266-43953301 |
|  | *NDP*-intron-2-2 | ACACGACGCTCTTCCGATCTCTCACTTAATGAAATTGTTTGCCTTTAGAGACTTGG | Reversed | chrX:43956461-43956496 |
|  | *NDP*-intron-1 | ACACGACGCTCTTCCGATCTGCTCTAGAAACCAATATTCTCCTCTCAAAATAACAT | Reversed | chrX:43958940-43958975 |
| Family 128 | *FZD4*-intron-1 | ACACGACGCTCTTCCGATCTAAAATGTAAATGTTAAGTCGCTGAAACTGCAGTACC | Forward | chr11:86953105-86953140 |
|  | *FZD4*-5' UTR | ACACGACGCTCTTCCGATCTACCCTTGTCTGCTCATTATATTATGTTTATTCCTCT | Forward | chr11:87003400-87003435 |
|  | *TMEM135*-intron-1 | ACACGACGCTCTTCCGATCTCCTTGGGGATGAAAACATATTTCACATTCACTATAC | Reversed | chr11:87053625-87053660 |
|  | *TMEM135*-intron-3 | ACACGACGCTCTTCCGATCTAGAAAGAAAAAATAGATAACTTGGACTTCTTCATCAAA | Reversed | chr11:87078572-87078609 |
| Family 131 | *LRP5*-5' UTR | ACACGACGCTCTTCCGATCTAGCCTAGAGGCACTTTTAGAGGAGTGTGTCTGTTT | Forward | chr11:68312028-68312062 |
|  | *LRP5*-intron1 | ACACGACGCTCTTCCGATCTAGAACATGTGTAGCGTTTCACGACTGCTTAAGTTT | Reverse | chr11:68313590-68313624 |
| Family 138 | *TSPAN12*-exon-4 | ACACGACGCTCTTCCGATCTCTATCACTGCTCCCTAATCTTGTGAACTGATTAAAA | Forward | chr7:120838682-120838717 |
|  | *TSPAN12*-exon-3 | ACACGACGCTCTTCCGATCTAAATAGCTGAGGGCAAAGTTACTTAAAATCAGCAAG | Forward | chr7:120839960-120839995 |
|  | *TSPAN12*-exon-2-1 | ACACGACGCTCTTCCGATCTGTTATCCCATGTAATTAATTAATGCTTAGCCATGCC | Forward | chr7:120856585-120856620 |
|  | *TSPAN12*-exon-2-2 | ACACGACGCTCTTCCGATCTAGTGAAACTTACTTACCCAAAAGAGCAGATTGAGGG | Forward | chr7:120856682-120856717 |
| Family 149 | *NDP*-exon-2-1 | ACACGACGCTCTTCCGATCTGACCCCATCTCTTAAAAAAAAATTATGGTGAATACC | Forward | chrX:43958551-43958586 |
|  | *NDP*-exon-2-2 | ACACGACGCTCTTCCGATCTATTGTAGAGGATGAAGTCTTTGAATGTAAGGCCTTT | Forward | chrX:43958621-43958656 |
|  | *NDP*-intron-1-1 | ACACGACGCTCTTCCGATCTAGAATTTAGTGTAGCTTAGCCTGAAAAGCATATGAT | Forward | chrX:43958869-43958904 |
|  | *NDP*-intron-1-2 | ACACGACGCTCTTCCGATCTCACTGTACCCAGCCTATAAATTAATTCTTTCGTAAT | Forward | chrX:43958947-43958982 |
